# The relationship between obstructive sleep apnea hypopnea syndrome and gastroesophageal reflux disease: a meta-analysis

**DOI:** 10.1007/s11325-018-1691-x

**Published:** 2018-07-09

**Authors:** Zeng-Hong Wu, Xiu-Ping Yang, Xun Niu, Xi-Yue Xiao, Xiong Chen

**Affiliations:** 1 Department of Otolaryngology, Union Hospital, Tongji Medical College, Huazhong University of Science and Technology, Wuhan, Hubei China; 2 Department of Obstetrics and Gynecology, Union Hospital, Tongji Medical College, Huazhong University of Science and Technology, Wuhan, China

**Keywords:** OSAHS, Gastroesophageal reflux disease, Meta-analysis

## Abstract

**Background:**

Obstructive sleep apnea hypopnea syndrome (OSAHS) means apnea and hypopnea caused by partial or complete obstruction of upper airway collapse during sleep. Gastroesophageal reflux disease (GERD) is believed to be associated with various manifestations in the otorhinolaryngology and has been found to be an additional risk factor for OSAHS.

**Aim:**

A meta-analysis was performed to identify the association between obstructive sleep apnea hypopnea syndrome and gastroesophageal reflux disease.

**Methods:**

To identify eligible original articles, we searched a series of computerized databases, including Medline via PubMed, EMBASE, Web of Science, and CNKI with a systematic searching strategy. The characteristics of each article and pooled odds ratios (ORs) with corresponding confidence intervals (CIs) were calculated and subgroup analysis was performed to analyze the source of heterogeneity.

**Results:**

A total of 2699 patients from seven articles were included in the meta-analysis. We identified a significant relationship between obstructive sleep apnea syndrome and gastroesophageal reflux disease, with a pooled OR of 1.75 (95% CI 1.18–2.59, *P* < 0.05). The pooled data was calculated under the random-effects model as a significant moderate heterogeneity was found among the meta-analysis.

**Conclusions:**

The meta-analysis showed that there was a significant correlation between obstructive sleep apnea hypopnea syndrome and gastroesophageal reflux disease.

## Introduction

Obstructive sleep apnea hypopnea syndrome (OSAHS) refers to apnea and hypopnea caused by partial or complete obstruction of upper airway collapse during sleep, usually accompanied by snoring, disturbed sleep architecture, decreased frequent oximetry values, daytime sleepiness, and inability to concentrate, that may further lead to multiple organ damages such as coronary heart disease, hypertension, type 2 diabetes mellitus, and dyslipidemia [1–3]. It is a prevalent sleep disorder, with at least 4% of middle-aged males and 2% of middle-aged females and among children estimated to be affected in population studies; the proportion can reach 3–12% [1]. Gastroesophageal reflux disease (GERD) is believed to be associated with various manifestations in the otorhinolaryngology and has been found to be an additional risk factor for OSAHS [4, 5]. At night, delayed gastric emptying, significantly delayed esophageal clearance, and marked reduction in upper esophageal sphincter pressure were observed. OSAHS is associated with a high frequency of GERD, because of increased intra-thoracic pressure, thus leading to acid reflux episodes [6, 7]. The refluxed content of the stomach in response to the development of OSAHS may cause upper airway inflammation and even obstruction. The association between OSAHS and GERD is controversial, and the previous studies are limited and contradictory.

In the past decade, a large number of studies were conducted try to find the mechanism of OSAHS and GERD and establish a causal relation between them. Treatment with continuous positive airway pressure (CPAP) can improve the sleep quality in OSAHS patients and has been shown to improve regurgitation [8]. A recent meta-analysis was designed to assess evidence of a relationship between the treatment of GERD with proton pump inhibitors (PPIs) and improvement in obstructive sleep apnea (OSA) and found that this way may improve the quality of sleep in night without any effect on apnea-hypopnea indices [9]. Furthermore, more studies are using polysomnography to document apnea index and 24-h pH monitoring to document the acid reflux and the prevalence of GERD in OSAHS patients. Kim et al. [10] found GERD was associated with more severe OSAHS and GERD symptoms were also associated with deteriorated sleep quality. However, this pathophysiological mechanism has been questioned in some studies as they find negative results. Kuribayashi et al. [11] reported that the occurrence of sleep-GERD and reflux esophagitis was not interrelated to the severity of OSA, and also not by negative intra-esophageal pressure due to OSA. So there is a controversy between OSAHS and GERD and need of more studies to conduct.

We thus performed a meta-analysis to grade the strength of evidence and systematically explore whether OSAHS correlates with GERD in the literature (supporting information: PRISMA Checklist) [12].

## Methods

### Literature search

Studies reporting the obstructive sleep apnea hypopnea syndrome and gastroesophageal reflux were identified for inclusion. To identify eligible original articles, we searched a series of computerized databases, including Medline via PubMed, EMBASE, Web of Science, and CNKI using the following key terms: “gastroesophageal reflux,” “reflux,” “obstructive sleep apnea hypopnea syndrome,” “OSAHS,” “obstructive sleep apnea,” “OSA” separated by the Boolean operator AND or OR. Articles were searched in the computerized databases up to January 2018, without limits of language. Reference lists from the resulting publications were used to identify further relevant publications. We screened the titles and abstracts of the identified studies, and articles that could contain data regarding GRED and OSAHS were evaluated the full article. Two authors (ZH Wu and XP Yang) independently searched for papers and screened the reference lists of retrieved articles to further identify potentially relevant publications. Discrepancies were resolved by consensus.

### Inclusion and exclusion criteria and data extraction

Original studies were carefully checked. There were no country restrictions. The inclusion criteria were (1) sleep disorders including OASHS; (2) studies in which all study group members exclude any possible predisposing factor (such as Barrett’s esophagus and asthma) that may be related to the development of their OSAHS; (3) studies that were restricted to humans, published in English and non-English, contained original data, and appeared in either abstract form or full text; (4) studies that clearly defined the study and control groups and the members, and observational data were available; and (5) studies that reported OSAHS patients with validated OSAHS (such as Epworth Sleepiness Score and Berlin questionnaire) questionnaire, polysomnography, and 24-h pH monitoring to document the acid reflux or anti-reflux treatment. The exclusion criteria were (1) case reports, non-English, abstracts, comments, review articles, duplicate publications, and editorials; (2) other treatment of obstructive sleep apnea (surgery or device); (3) studies that focus on laryngopharyngeal reflux (LPR) disease.

### Data extraction

Information was collected for each publication concerning the author’s name, publication year, study design, age, method of reflux evaluation or OSAHS evaluation, and study and control group criteria.

### Risk of bias and statistical analysis

We used the PRISMA statement [13] to assess individual study quality and the risk of bias. Meta-analysis was performed using Review Manager 5.3 (Cochrane Collaboration, Copenhagen, The Nordic Cochrane Centre). Random-effects model was applied, depending on the *P* value of the chi-squared statistic when *P* was < 0.05. Higgins *I*^2^ test were used to assess the heterogeneity. An *I*^2^ value between 25 and 50% was considered as low heterogeneity, an *I*^2^ value between 50 and 75% as moderate heterogeneity, and an *I*^2^ value > 75% as high heterogeneity. An *I*^2^ value < 25% was considered homogeneous. When *I*^2^ value > 50%, the random-effects model was applied to combine effect size and when *I*^2^ value < 50% the fix-effects model was applied to combine effect size. We also sought to perform subgroup analysis to determine the sources of heterogeneity. The pooled odds ratios (ORs) of different articles and corresponding 95% confidence intervals (CIs) were used to estimate the relationship between OSAHS and GERD. The sensitivity analysis was repeated to assess the effects of individual study on pooled estimates by removing individual study.

## Results

### Study selection

**Fig. 1:**
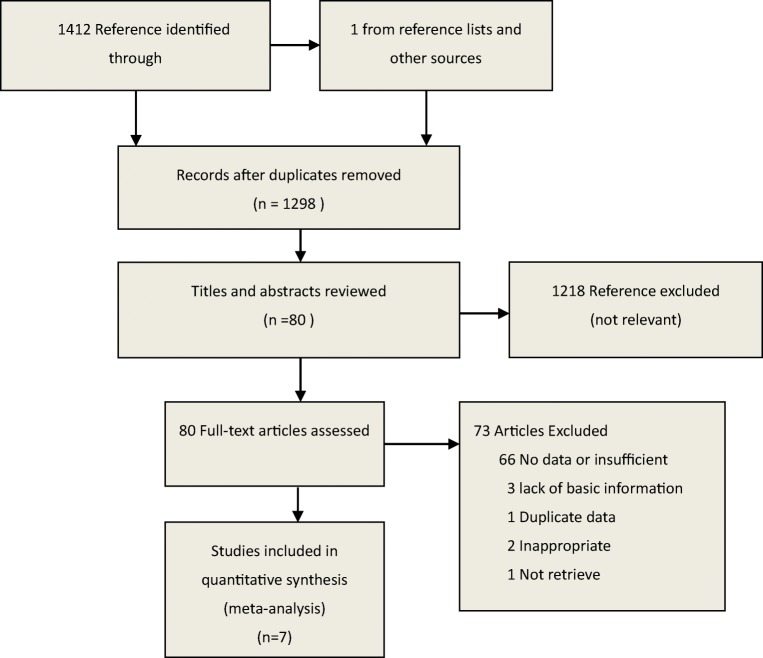
Search strategy to identify articles on the relationship between obstructive sleep apnea hypopnea syndrome and gastroesophageal reflux disease

Our search strategy identified 1412 potentially relevant articles from electronic databases and 1 from reference lists and other sources. After excluding duplicates, 1298 records remained. Reading the titles and abstracts of these 1298 references led us to exclude 1218 articles that did not meet the inclusion criterion. After reading the full text of the remaining 80 articles as possibly reporting the relationship between OSAHS and GERD, 66 were excluded because the lack of sufficient data; 2 study were excluded because the outcome measure is inappropriate; 3 lacked basic information; 1 study was duplicate and we could not retrieve 1 study for full text. Ultimately, seven eligible articles were identified [14–20]. The selection process was shown in Fig. 1 and the detailed information of each study was listed in Table 1.

**Table 1:** Description of included studies

Source, year	Country	Mean age, year	Sample size	Study design	Reflux criteria or OSAHS criteria	Study and control group
Cummings et al. 2013 [14]	USA	57	233	Case–controls	Esophagogastroduodenoscopy, colonoscopy, Berlin Questionnaire	GERD versus non-GERD
XIAO et al. 2012 [15]	China	40	53	Cross-sectional	24-h MII–pH monitoring, polysomnography	OSAHS versus non-OSAHS
You et al. 2014 [16]	Korea	55	849	Cross-sectional	Reflux questionnaire, Berlin Questionnaire	GERD versus non-GERD
Basoglu et al. 2014 [17]	Turkey	NA	1104	Cross-sectional	Validated GERD questionnaire, polysomnography	OSAHS versus non-OSAHS
Vela et al. 2014 [18]	USA	NA	158	Cross-sectional	Validated GERD questionnaire, polysomnography	GERD versus non-GERD
Chen et al. 2016 [19]	China	NA	60	Cross-sectional	Polysomnography	GERD versus non-GERD
Lv et al. 2017 [20]	China	NA	84	Cross-sectional	Validated GERD questionnaire, 24-h pH monitoring	OSAHS versus non-OSAHS

*GERD*, gastroesophageal reflux disease; *MII–pH*, multichannel intraluminal impedance–pH

The meta-analysis consisted with a total sample size of 2699 included one case–control study and six cross-sectional studies. Two articles used 24-h pH monitoring to evaluate the relationship between OSAHS and GERD in cases and controls. Four articles used polysomnography to evaluate the apnea-hypopnea indices.

### Meta-analysis results

**Fig. 2:**
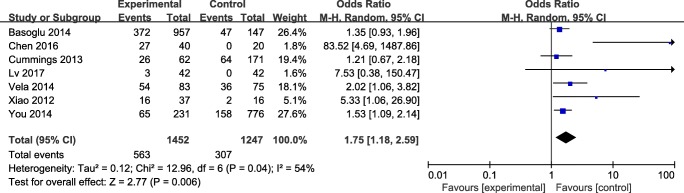
Relationship of OSAHS and GERD. The results indicated that OSAHS was significantly associated with risk of GERD (OR = 1.75, 95% CI = 1.18–2.59)

The forest plot result for association of OSAHS with GERD is shown in Fig. 2. We identified a significant relationship between OSAHS and GERD, with a pooled OR of 1.75 (95% CI 1.18–2.59, *P* < 0.05). The pooled data was calculated under the random-effects model as a significant moderate heterogeneity was found among the studies.

### Subgroup analysis

#### Study and control group

##### GERD versus non-GERD

**Fig. 3:**
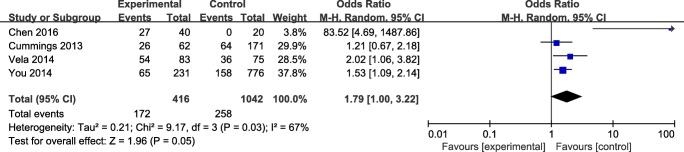
The forest plot of GERD versus non-GERD subgroup analysis

The results was significant, with a corresponding value of 1.79 (95% CI 1.00 to 3.22, *P* < 0.05) under the random-effects model. The forest plot about the subgroup analysis is showed in Fig. 3.

##### OSAHS versus non-OSAHS

**Fig. 4:**
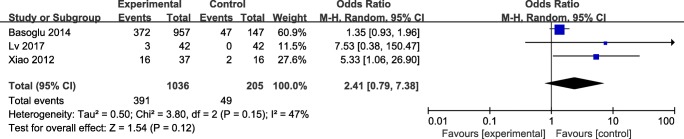
The forest plot of OSAHS versus non-OSAHS subgroup analysis

The results was significant, with a corresponding value of 1.53 (95% CI 1.07 to 2.08, *P* > 0.05) under the fixed-effects model. The forest plot about the subgroup analysis is showed in Fig. 4**.**

#### Method for OSAHS

##### Polysomnography

**Fig. 5:**
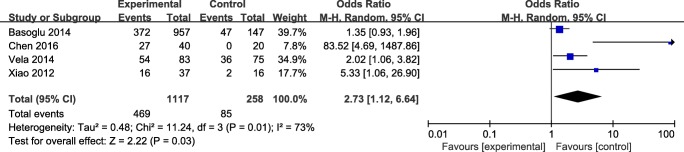
The forest plot of polysomnography subgroup analysis

The results was significant, with a corresponding value of 2.73 (95% CI 1.12 to 6.64, *P* < 0.05) under the random-effects model. The forest plot about the subgroup analysis is showed in Fig. 5.

##### Berlin Questionnaire

**Fig. 6:**
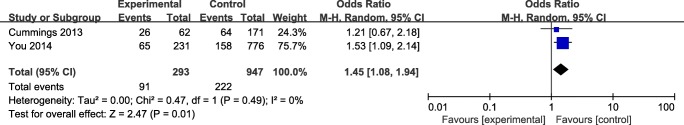
The forest plot of Berlin Questionnaire subgroup analysis

The results was significant, with a corresponding value of 1.44 (95% CI 1.08 to 1.93, *P* < 0.05) under the fixed-effects model. The forest plot about the subgroup analysis is showed in Fig. 6**.**

#### Reflux questionnaire

**Fig. 7:**
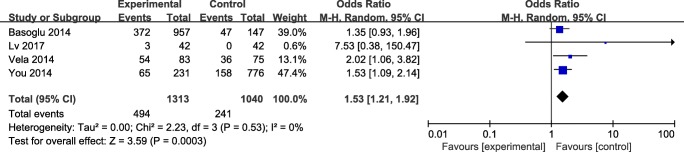
The forest plot of reflux questionnaire subgroup analysis

The results was significant in reflux questionnaire subgroup analysis, with a corresponding value of 1.53 (95% CI 1.22–1.93, *P* > 0.05) under the fixed-effects model. The forest plot about the subgroup analysis is showed in Fig. 7**.**

#### 24-h pH monitoring

**Fig. 8:**
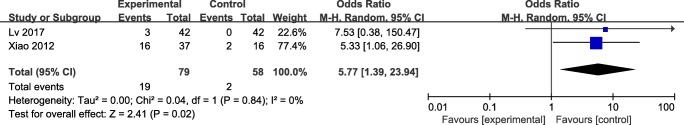
The forest plot of 24-h pH monitoring subgroup analysis

The results was significant in 24-h pH monitoring subgroup analysis, with a corresponding value of 5.83(95% CI 1.41–24.15, *P* > 0.05) under the fixed-effects model. The forest plot about the subgroup analysis is showed in Fig. 8. The results showed that the gastroesophageal reflux materials may play an important role in OSAHS pathogenesis.

### Sensitivity analysis

The coupled forest plots show moderate heterogeneity (*I*^2^ = 54%) and when we removed Chen’s study, the results dramatically influenced the pooled results (*I*^2^ decreased from 54 to 3%) in the meta-analysis with the random-effects model. The outcome of sensitivity analysis showed that the pooled ORs ranged from 1.52 (95% CI 1.22–1.89) to 2.10 (95% CI 1.19–3.69). Moreover, in subgroup analyses, *I*^2^ = 67% for GERD versus non-GERD; *I*^2^ = 47% for OSAHS versus non-OSAHS; *I*^2^ = 0% for Berlin Questionnaire; *I*^2^ = 73% for polysomnography; *I*^2^ = 0% for reflux questionnaire; and *I*^2^ = 0% for 24-h pH monitoring. However, in GERD versus non-GERD subgroup, the results dramatically are influenced by removed Chen’s study (*I*^2^ decreased from 67 to 0%), and in OSAHS versus non-OSAHS subgroup, when we removed Basoglu’s study, the *I*^2^ decreased from 47 to 0%.

### Risk of bias

**Fig. 9:**
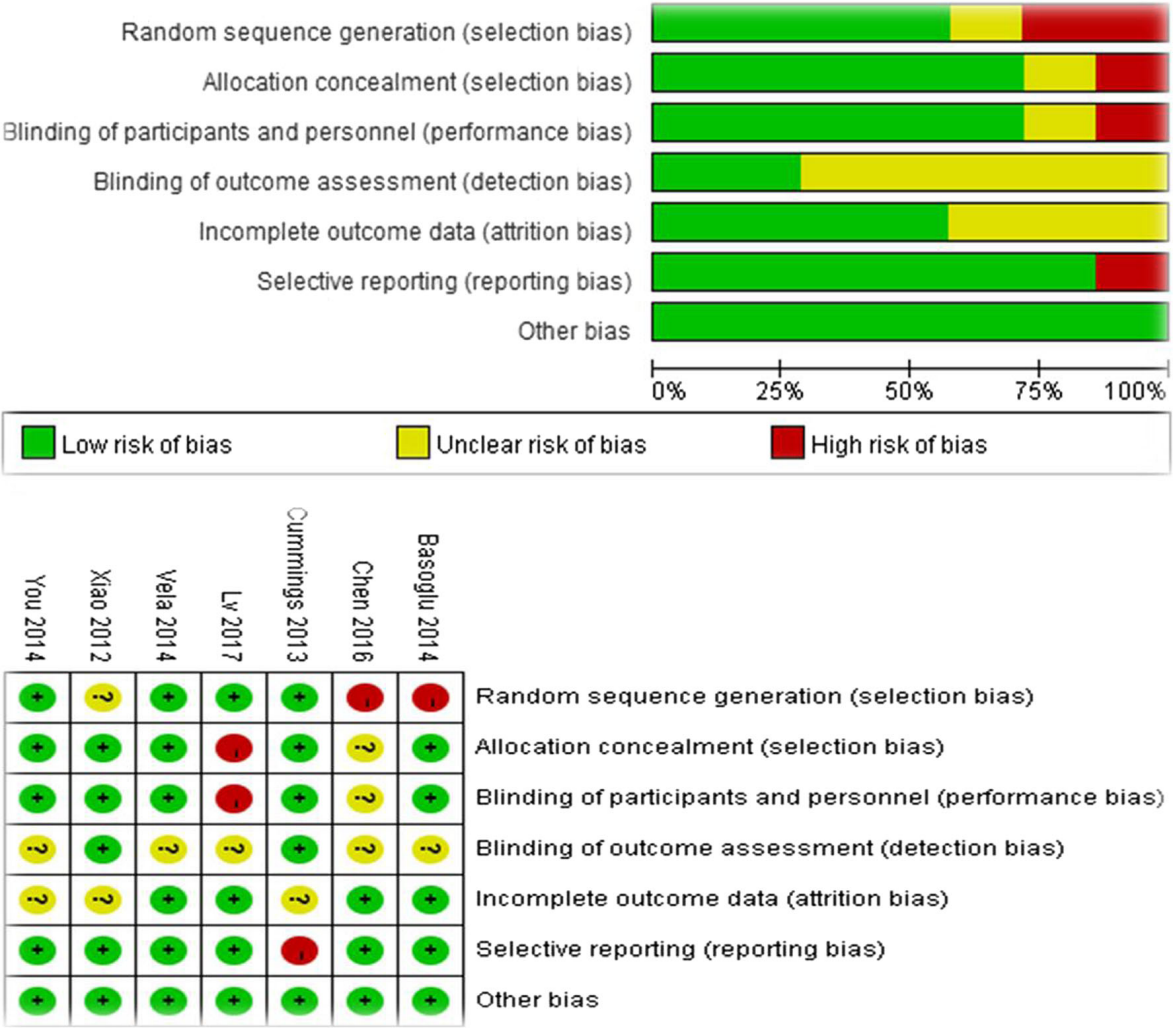
Risk of bias summary and graph: review of authors’ judgements about each risk of bias item for each included study. The PRISMA guidelines require an analysis of potential biases, which would lead to under- or overestimation of the true intervention effect. Referring to the PRISMA guidelines, the authors judged the risk of bias (low, unclear, high risk of bias) for the following items for each included study: selection bias, blinding of the participants and personnel, detection bias, attrition bias, reporting bias, and other bias. Shown are the authors’ judgments about each risk of bias item for each study (upper part) and as percentages across all included studies (lower part)

The trim and fill method indicated that there is no study needed to be statistically corrected for funnel plot asymmetry. The methodological quality of each included study is shown in Fig. 9**.**

## Discussion

This meta-analysis suggested that OSAHS was significantly associated with increased risk of GERD (OR = 1.75, 95% CI 1.18–2.59, *P* < 0.05). The pooled results were credible as the subgroups were analyzed. Although the value of *I*^2^ = 54% (*I*^2^ > 50, *P* < 0.05), it indicated that there existed moderate heterogeneity among the studies. But we performed the subgroup analysis to determine the sources of heterogeneity so the results of meta-analysis could represent the true relationship between OSAHS and GERD. Meanwhile, sensitivity analysis showed that after any individual study was omitted or the random-effects model was converted to a fixed-effects model, the overall results and conclusions still exist. Therefore, we have strong confidence to believe the result of our meta-analysis that strong association between OSAHS and GERD.

Recent researches have reported that OSAHS and symptomatic in GERD are closely related. However, the exact causative relationship between them remains contradictory and this is the first meta-analysis to approve it. In Basoglu et al. [17] study, it was shown that 38.9% of OSAHS patients accompanied GERD, and the prevalence of GERD in OSAHS patients was fairly increased compared to the general population. Soren et al. [21] reported that the episodes of acidification were very common in patients with sleep apnea and usually associated with pressure and respiratory events. Our findings and these conclusions are consistent; the summary OR was 1.75 (95% CI 1.18–2.59, *P* < 0.05) in our meta-analysis under the random-effects model. Thus, we can conclude that GRED may play a very important role in the pathophysiology of OSAHS. OSAHS may be related to age, gender, BMI, alcohol, or Barrett’s esophagus (BE) in GRED patients. Ju et al. [22] evaluated 564 subjects who were investigated in the sleep laboratory with a GERD questionnaire and found that GERD was more common in female subjects. In the past years, some studies pay attention to the association between OSAHS and Barrett’s esophagus because the aggravated reflux might result in an increased risk of this disease. Barrett’s esophagus is a precursor to esophageal adenocarcinoma and it was reported that GRED and obesity (especially in abdominal adiposity) are the two strongest risk factors [23, 24]. Thus, we did not included Barrett’s esophagus data in our meta-analysis of studies.

Usually, polysomnography is the standard diagnostic modality for OSAHS, but this procedure requires an overnight evaluation, so more studies have used questionnaires to evaluate patients’ sleep quality (such as the Berlin Questionnaire) [25]. However, these questionnaires cannot allow us to obtained objective apnea-related parameters. The summary OR was 2.73 (95% CI 1.12 to 6.64, *P* < 0.05) for polysomnography subgroup analysis with the random-effects model and the summary OR was 1.44 (95% CI 1.08 to 1.93, *P* < 0.05) for Berlin Questionnaire subgroup analysis with the fixed-effects model. The subgroup analysis results indicated that GRED had a strong association with OSAHS. The standard diagnostic of GERD is 24-h dual-probe esophageal pH monitoring, and it can record episodes of not only laryngopharyngeal reflux but also gastroesophageal reflux. But in our meta-analysis, we only contain the data from gastroesophageal reflux. Many of patients (OSAHS in particular) maybe did not accept this application, as this is an invasive method. So few studies used this application assess the relationship between the OSAHS and GRED patients. We can only include two studies in our meta-analysis, and the summary OR was 5.83(95% CI 1.41–24.15, *P* > 0.05) for 24-h pH monitoring subgroup analysis under the fixed-effects model. These results supported that GERD participation in OSAHS pathogenesis.

From this meta-analysis results, we may arrival at a conclusion that the GERD participation in OSAHS pathogenesis. On one hand, for patients with GERD, we can try anti-reflux treatment (such as proton pump inhibitors) and lifestyle modifications (such as dietary, weight control, and no alcohol). On the other hand, we may also suggest that OSAHS patients be evaluated for GERD before undergoing surgical treatment or CPAP treatment. As an otorhinolaryngologist, if we suspected OSAHS patients with GERD signs and symptoms (such as frequent throat clearing, reflux laryngitis, sore throat and posterior laryngitis with hoarseness, chronic cough, and even laryngeal and subglottic stenosis [26]), fiberoptic laryngoscopy or 24-h dual-probe esophageal pH monitoring should be performed. Thus, we can identify high-risk patients and treated GRED disease first to lower unnecessary treatment for OSAHS.

There are some potential limitations in the meta-analysis that should be squared up when interpreting the results of our study. Firstly, the sample size was relatively not enough and may affect the accuracy of our results and much large-scale studies should be performed to convince it. Secondly, although we try to explore the source of heterogeneity by subgroup analysis in our study, we could not successful explore heterogeneity from other aspects because of the insufficient clinical data and the limited studies. Thirdly, the results may also be biased by different measurement techniques to diagnosis OSAHS (polysomnography/Berlin Questionnaire) or GERD (reflux questionnaire/24-h pH monitoring). Additionally, as most of the included study population was adult, the results may be different in children population. So our results should be interpreted with caution and need further researches. Despite there are limitations, our analysis shows a strong and clear association between OSAHS and GERD.

## Conclusions

This meta-analysis provided direct evidence of the GERD participation in OSAHS pathogenesis and suggested that in treatment of OSAHS, the GERD disease should not be neglected in clinical practice.
